# Molecular pathways underpinning ethanol-induced neurodegeneration

**DOI:** 10.3389/fgene.2014.00203

**Published:** 2014-07-15

**Authors:** Dan Goldowitz, Alexandre A. Lussier, Julia K. Boyle, Kaelan Wong, Scott L. Lattimer, Candis Dubose, Lu Lu, Michael S. Kobor, Kristin M. Hamre

**Affiliations:** ^1^Centre for Molecular Medicine and Therapeutics, Child and Family Research Institute – Department of Medical Genetics, University of British ColumbiaVancouver, BC, Canada; ^2^Department of Anatomy and Neurobiology, University of Tennessee Health Science CenterMemphis, TN, USA; ^3^Human Early Learning Partnership, School of Population and Public Health, University of British ColumbiaVancouver, BC, Canada

**Keywords:** QTL, apoptosis, hippocampus, cerebral cortex, chromosome modifications, histone marks

## Abstract

While genetics impacts the type and severity of damage following developmental ethanol exposure, little is currently known about the molecular pathways that mediate these effects. Traditionally, research in this area has used a candidate gene approach and evaluated effects on a gene-by-gene basis. Recent studies, however, have begun to use unbiased approaches and genetic reference populations to evaluate the roles of genotype and epigenetic modifications in phenotypic changes following developmental ethanol exposure, similar to studies that evaluated numerous alcohol-related phenotypes in adults. Here, we present work assessing the role of genetics and chromatin-based alterations in mediating ethanol-induced apoptosis in the developing nervous system. Utilizing the expanded family of BXD recombinant inbred mice, animals were exposed to ethanol at postnatal day 7 via subcutaneous injection (5.0 g/kg in 2 doses). Tissue was collected 7 h after the initial ethanol treatment and analyzed by activated caspase-3 immunostaining to visualize dying cells in the cerebral cortex and hippocampus. In parallel, the levels of two histone modifications relevant to apoptosis, γH2AX and H3K14 acetylation, were examined in the cerebral cortex using protein blot analysis. Activated caspase-3 staining identified marked differences in cell death across brain regions between different mouse strains. Genetic analysis of ethanol susceptibility in the hippocampus led to the identification of a quantitative trait locus on chromosome 12, which mediates, at least in part, strain-specific differential vulnerability to ethanol-induced apoptosis. Furthermore, analysis of chromatin modifications in the cerebral cortex revealed a global increase in γH2AX levels following ethanol exposure, but did not show any change in H3K14 acetylation levels. Together, these findings provide new insights into the molecular mechanisms and genetic contributions underlying ethanol-induced neurodegeneration.

## INTRODUCTION

Alcohol exposure during development induces a number of lasting physiological changes that result in a host of abnormalities in brain function. It has been consistently shown that the type and severity of ethanol-induced changes can be modulated by the genetics of the individual as shown in studies in both animals (Goodlett et al., 1989; Gilliam and Irtenkauf, 1990; Boehm et al., 1997; Gilliam et al., 1997; Ogawa et al., 2005; Downing et al., 2009) and humans (Christoffel and Salafsky, 1975; Chasnoff, 1985; Streissguth and Dehaene, 1993; Riikonen, 1994). As the mechanisms underlying this phenomenon remain unknown, rigorous examination of the relationship between ethanol exposure, its key physiological targets, and genetic variation will enable the identification of the molecular underpinnings of the resulting damage.

The central nervous system (CNS) is especially susceptible to developmental defects following alcohol exposure, with ethanol causing aberrant mitosis and cell migration, as well as alterations in neuronal process outgrowth and connectivity (as reviewed in Sulik et al., 1988; Kumada et al., 2007; Sadrian et al., 2013). However, one of the most common ethanol-induced alterations is cell death including apoptotic cell death. Cell death occurs in a time and dose-dependent fashion although there are windows of time when specific cell populations are particularly vulnerable to ethanol-induced cell death. For example, the work of Sulik and colleagues demonstrates that developing neuroblasts are particularly vulnerable to ethanol-induced cell death shortly after neural tube closure (Dunty et al., 2001, 2002) while Olney et al. (2002a,b), Dikranian et al. (2005) demonstrated that the more mature neurons in the developing cerebral cortex and other forebrain structures are particularly vulnerable to alcohol during the early postnatal period in mice. In the present study, ethanol was administered to mice postnatally during the time of the brain growth spurt (Dobbing, 1974). During the brain growth spurt, neurons are completing migration and actively establishing connections (Dobbing, 1974). The equivalent stage of brain growth in humans begins during the third trimester and continues the first 1–2 years after birth (Dobbing and Sands, 1979).

The genetic contributions to a phenotype are often explored using knockout animals (e.g., de Licona et al., 2009; Noel et al., 2011). While this strategy is effective in establishing the function of a specific gene, it does not reflect the spectrum and complexity of variation observed across a population. To circumvent this drawback, we have harnessed the natural variation present within mice through the use of BXD recombinant inbred strains, generated by crossing the C57BL/6J and DBA/2J strains (Morse et al., 1979; Peirce et al., 2004).

Furthermore, genetic background also contributes to the distribution of epigenetic patterns established during early development (Weng et al., 1995; Padjen et al., 2005; Schilling et al., 2009). As histone modifications and DNA methylation also respond to various environmental and cellular cues, epigenetic marks may provide a link between genetic variation and susceptibility to ethanol-induced cell death (Meaney, 2010; Kobor and Weinberg, 2011). Previous studies have investigated the effect of different exposure paradigms on histone modifications in the brain, finding that chromatin structure responds to various teratogens during development (Cronican et al., 2013; Jordi et al., 2013; Luo et al., 2014). Recent evidence also shows that acute ethanol exposure alters dimethylation levels on lysine 9 and 27 of histone 3, which partially mediate ethanol’s teratogenic effects in the brain (Subbanna et al., 2013).

In the present study, a high dose of ethanol was administered to mice during the brain growth spurt at postnatal day 7 (P7) (Dobbing, 1974). Previously, strain differences in levels of ethanol-induced cell death were observed following early prenatal ethanol exposure (Chen et al., 2011) and the current study expanded this to neonatal exposure. In order to identify the contribution of the genetic background to variable susceptibility to ethanol-induced cell death, activated caspase-3 immunostaining was performed on the hippocampus and different layers of the cerebral cortex in BXD strains. Quantitative trait locus (QTL) analysis was performed to identify chromosomal locations involved in the observed differences. Finally, as an initial analysis of chromatin-based modifications in ethanol-induced apoptosis, the effect of alcohol exposure on the levels of two histone modifications, phosphorylated H2A.X and acetylated lysine 14 of histone H3, was investigated using protein blots.

## MATERIALS AND METHODS

### ANIMALS

**FIGURE 1:**
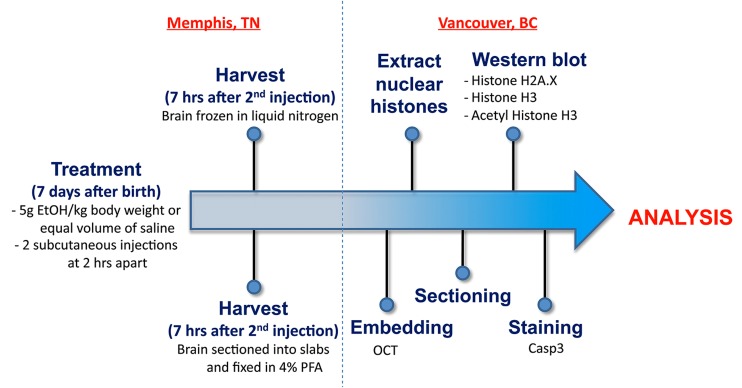
**An overview of the workflow from generation of postnatal mice through to staining and analysis**.

**Figure 1** illustrates the experimental flow from the generation of mice through to the analysis. All animals were maintained at the University of Tennessee Health Science Center (UTHSC). Mice were maintained on a 12:12 light:dark cycle and given food and water ad libitum. The BXD strains, generated by crossing the C57BL/6J (B6) and DBA/2J (D2) parental strains and inbreeding the resulting offspring for over 20 generations (Morse et al., 1979; Peirce et al., 2004), as well as the B6 and D2 parental strains, were used in the current analyses. All experiments were conducted with approval of the Institutional Animal Care and Use Committee at UTHSC. All adult mice used to generate the neonates were a minimum of 90 days of age.

Timed matings were used to generate offspring used in this study. Males and females were mated for 4 h starting between 9 and 10 a.m. daily. After the 4 h, the females were removed and checked for the presence of a vaginal plug. The presence of a vaginal plug was termed day 0 of gestation. For the neonatal mice, the day of birth was recorded and animals were exposed to ethanol or control solution on postnatal day 7 (P7).

### ETHANOL EXPOSURE AND TISSUE COLLECTION

The protocol of Olney et al. (2002a) was followed. Postnatal day 7 (P7) mice were given ethanol (20% v/v in saline) via subcutaneous injection. The total dose of ethanol was 5.0 g/kg given in two injections of 2.5 g/kg separated by 2 h. Controls were given isovolumetric saline. A maximum of one male and one female in each group from each litter were used and multiple litters were evaluated from each strain. Animals were sacrificed 7 h after the initial injection.

The P7 neonates used for cell count analysis were lightly anesthetized on ice and sacrificed by decapitation. Each brain was dissected from the skull and placed into a tissue chopper where 1 mm slabs were cut. Each slab was fixed overnight in 4% PFA and changed to buffer for tissue processing. For epigenetic analyses, the brain was dissected from the skull and microdissected into various brain regions including the hippocampus and cortex. Each region was placed into a separate microfuge tube, frozen in liquid nitrogen and stored at –80°C until processing.

### TISSUE PROCESSING

One slab from each brain, corresponding approximately to the region Bregma –1.955 to –2.48 mm, was picked for sectioning in a cryostat and cryoprotected using 30% sucrose in PBS and embedded in OCT (Sakura). Coronal sections were cut at 16 μm thickness in a cryostat and directly mounted on glass slides (SuperFrost Plus, Fisher Scientific).

### DETECTION OF CELL DEATH

For detection of apoptotic cells in P7 brains, we performed immunohistochemistry using an antibody specific for activated caspase-3 (Abcam). Tissue sections were put in boiling 10 mM citrate buffer for 6 min and then treated with 0.3% hydrogen peroxide to quench endogenous peroxidases. Sections were incubated in blocking solution, containing 30% bovine serum albumin (1:100, Sigma-Aldrich), normal goat serum (1:10, Bethyl Laboratories), and triton X-100 (1:100, Fisher Scientific) in PBS, for 20 min, after which they were incubated in primary antibody at a 1:1000 dilution at room temperature overnight. The sections were rinsed and incubated for 1 h with biotinylated goat anti-rabbit IgG (ABC Elite Kit, Vector Laboratories). Sections were next incubated with Avidin and Biotinylated horseradish peroxidase (Vector Laboratories) for 30 min at room temperature. Immunostaining was visualized using Diaminobenzidine and tissues were counterstained with methyl green.

### QUANTIFICATION OF CELL DEATH

The tissues were examined with a Zeiss fluorescence microscope and photomicrographs were taken with Axio Vision software (version 4.6). Area measurements were performed using ImageJ software (National Institutes of Health, version 1.43s). To quantify cell death, three sections from each sectioned slab were chosen, each separated by five sections. A 200 μm wide band of the CA1 region that was just distal to the CA1/2 border of the hippocampus and overlying cortex were chosen for quantification. The areas of CA1 or cortex that fell within the band were measured for caspase 3-positive cells and total cell number. Cortical layers were determined by visual assessment of cell morphology. To obtain a measure of cell death, the total number of cells within the region of interest in each section was estimated by obtaining a count of the number of cells within a 50 μm × 50 μm bin and extrapolating over the total area. The total number of stained cells within the 200 μm wide band was counted to determine a percentage of activated caspase 3-positive cells.

### QTL ANALYSIS

Cell death data were registered in GeneNetwork, an open access online database containing BXD genomic information (http://www.genenetwork.org). Genome-wide interval mapping of QTLs regulating cell death was performed using WebQTL, a module of GeneNetwork. The likelihood ratio statistic (LRS) was computed to assess the strength of genotype–phenotype association of the genomic scans. Permutation test of 2000 permutations was computed to establish the significant and suggestive thresholds where the LRS values corresponded to a genome-wide *p*-value of 0.05 and 0.63, respectively. A significant QTL is referred to as a chromosomal region with LRS score equal to or above the genome-wide significant level (*p* = 0.05). A suggestive QTL is a region of the chromosome with LRS score equal or above the genome-wide suggestive level (*p* = 0.63).

### PROTEIN BLOT ANALYSIS OF HISTONE MARKS

Nuclear histones were extracted from the cerebral cortex of male P7 mice (three control and four ethanol-treated) using previously described methods (Rumbaugh and Miller, 2011). Histones were loaded onto 15% SDS-polyacrylamide gels and separated by electrophoresis. Proteins were transferred onto nitrocellulose membranes and blocked with 5% milk for 2 h at room temperature. Membranes were incubated for 2 h with rabbit primary antibody at room temperature, followed by 16 h incubation at 4°C with mouse primary antibody. They were then incubated with secondary antibodies against mouse and rabbit (1/15,000) for 1 h at room temperature. Membranes were washed for 3 × 5 min between incubations with 0.1% Tween-20 Tris-buffered saline (TBST). Bands were imaged using the Li-Cor Odyssey scanner.

The antibodies used were as follows: 1/1000 rabbit polyclonal to histone H2A.X (ab10475, Abcam), 1/1000 mouse monoclonal to H2A.X (phospho-S139) (ab18311, Abcam), 1/2000 mouse monoclonal to histone H3 (ab10799, Abcam), 1/2000 rabbit polyclonal antibody to acetyl-histone H3 (Lys14) (06-911, Millipore), IRDye^®^ 800CW conjugated Goat (polyclonal) anti-mouse IgG (926-32210, Li-Cor Biosciences), IRDye^®^ 680 conjugated Goat (polyclonal) anti-rabbit IgG (926-32221, Li-Cor Biosciences).

### QUANTIFICATION OF PROTEIN BLOTS

Using ImageStudioLite software (LiCor, Lincoln, NE), boxes were placed around each band of interest, which returned values of raw intensity. Background was removed from raw values using the median correction function to obtain the signal intensity for each protein band. γH2A.X signal intensity was normalized to total H2AX to obtain the relative ratio of γH2A.X/H2A.X for each sample and acetylated H3 (Lys14) signal was normalized to total H3 to obtain the relative ratio of H3K14ace/H3. Statistically significant differences (*p* < 0.05) were identified using Student’s *t*-test in Graphpad Prism 6.

## RESULTS

The following study was designed to identify genetic differences in susceptibility to ethanol-induced cell death, as well as chromatin-based mechanisms that could modulate alcohol’s teratogenic effect. As shown in **Figure 1**, mice from BXD strains were exposed to a high ethanol dose at P7 by subcutaneous injection. Brains were harvested and specific regions of the hippocampus and cerebral cortex analyzed for the level of ethanol-induced cell death. In parallel, whole brain regions were dissected from other samples for histone extractions and subsequent analyses of salient chromatin modifications.

### GENETIC BACKGROUND ALTERED LEVELS OF ETHANOL-INDUCED CELL DEATH

In spite of the similarity observed between B6 and D2 progenitor strains, considerable differences in susceptibility to apoptosis were observed between various BXD recombinant inbred strains following ethanol treatment at P7.

**FIGURE 2:**
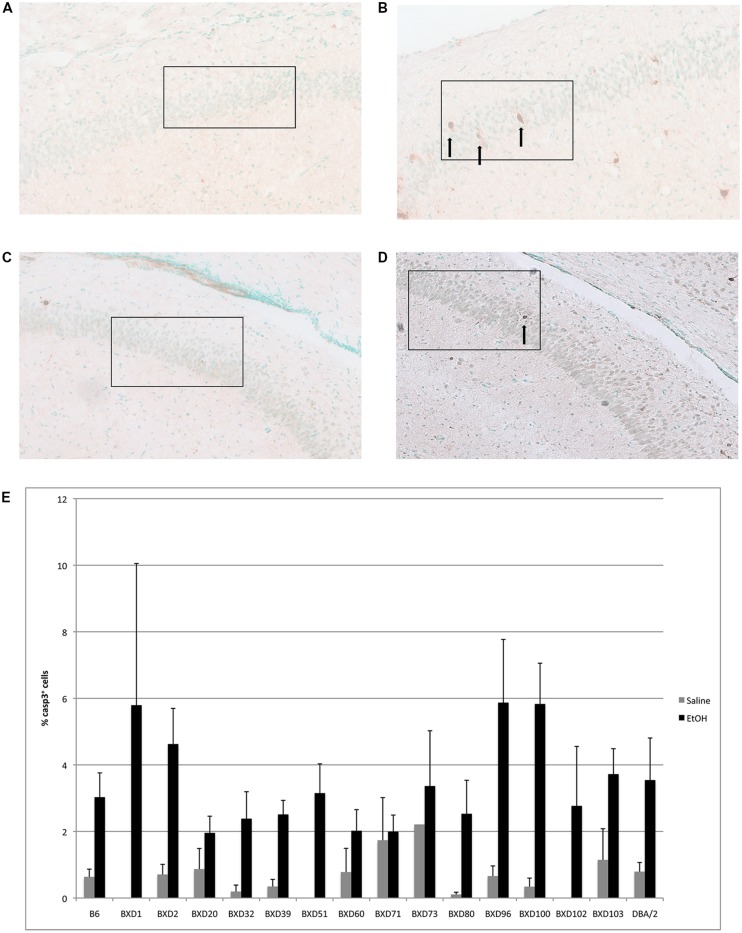
**(A)** Cell death is shown in the CA1 region of resistant (BXD20) and **(B)** relatively susceptible (BXD96) strains. **(C,D)** Saline controls for BXD20 and BXD96, respectively. Boxes indicate the areas analyzed, arrows point to dying cells. **(E)** Cell death in CA1 is plotted as mean + SEM.

In the CA1 region of the hippocampus, mean levels of caspase-3 positive cells following alcohol exposure varied between 2 and 6% of total cells within the analyzed area (**Figure 2**). Out of fourteen different lines, four exhibited cell death levels greater than 4% (BXD 1, 2, 96, 100), which were flagged as higher susceptibility backgrounds for subsequent analyses. In contrast, three strains (BXD 20, 60, 71) only displayed 2% or less mean apoptosis and were thus labeled as low vulnerability strains. Caspase-3 immunostaining of the hippocampal CA1 region in high (BXD96) and low (BXD20) susceptibility strains is illustrated in **Figure 2**.

**FIGURE 3:**
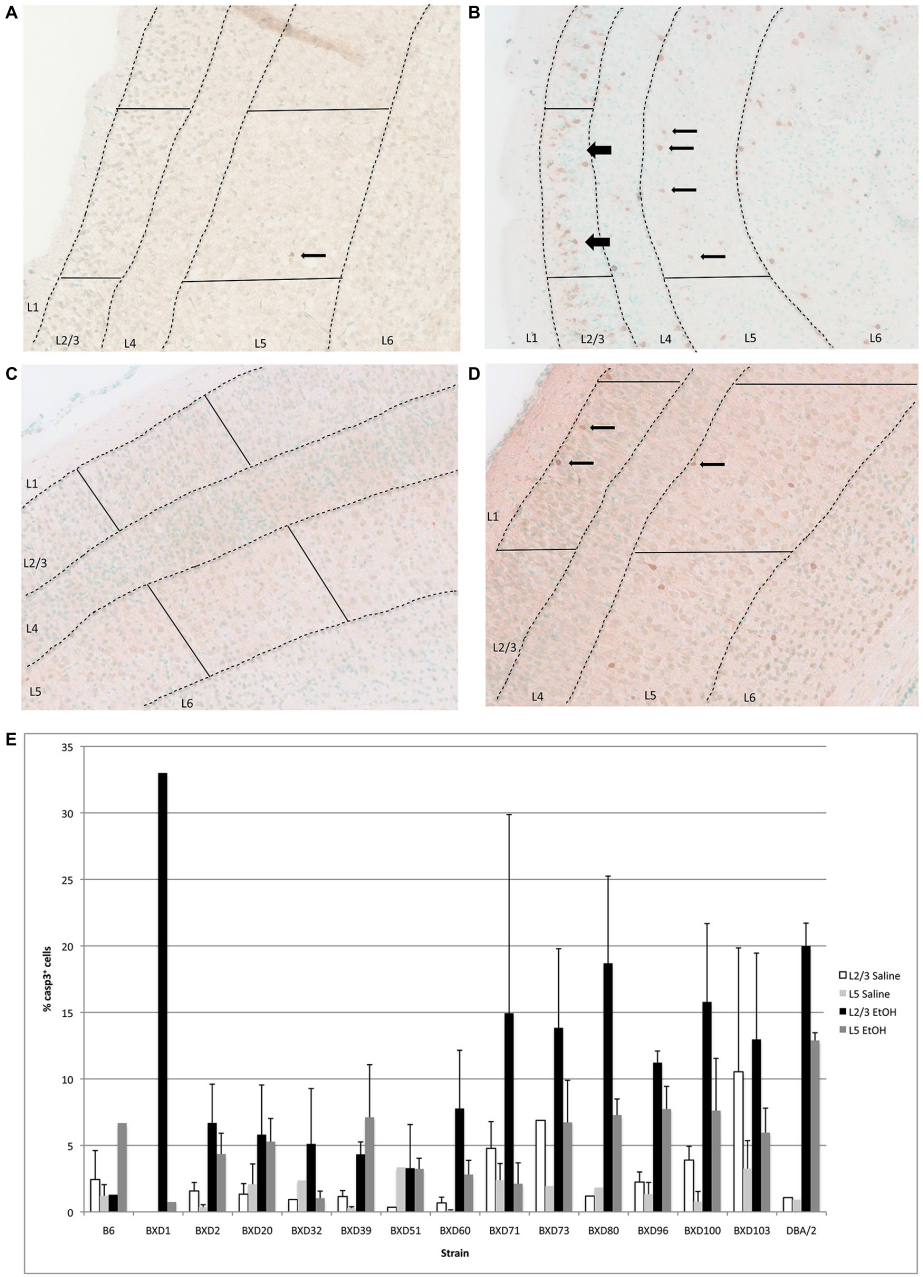
**(A)** Cell death is shown in the cortex overlying the hippocampus of a region of a relatively resistant (BXD20) strain, and **(B)** a region of a relatively susceptible (BXD80) strain. **(C,D)** Saline controls for BXD20 and BXD80, respectively. Boxes highlight the area analyzed, dashed lines demarcate Layers 2/3 and 5, and arrows point to dying cells. **(E)** Cell death in cortex is plotted for Layers 2/3 and 5 as mean + SEM.

In the cerebral cortex, ethanol-induced apoptosis was mainly localized to Layers 2/3, and 5 (**Figure 3**), with the highest levels of neurodegeneration occuring in Layer 2/3. Cell death occurred mainly in the superficial portion of Layers 2/3, while Layer 5 displayed a more homogeneous pattern of apoptosis. This specific localization was maintained across strains showing differential cell death levels. However, mean levels of apoptosis differed between genetic backgrounds (**Figure 3B**), ranging from below 5–20% of all cells within the analyzed area. Out of fourteen different strains, three (BXD 71, 80, 100) exhibited mean levels of cell death greater than 15% in Layer 2/3, and were identified as high vulnerability strains. Alternatively, three lines (BXD 32, 39, 51) showed 5% or less caspase-3 positive cells, and were flagged as low susceptibility backgrounds. In almost all cases, the percentage of cell death in Layer 5 was lower than Layer 2/3. However, the trend was similar between strains, where higher or lower cell death in a given line was observed in both regions. Caspase-3 immunostaining of the cerebral cortex in high (BXD80) and low (BXD20) vulnerability strains is illustrated in **Figure 3A**.

### IDENTIFICATION OF AN ETHANOL-SUSCEPTIBILITY QUANTITATIVE TRAIT LOCUS

**FIGURE 4:**
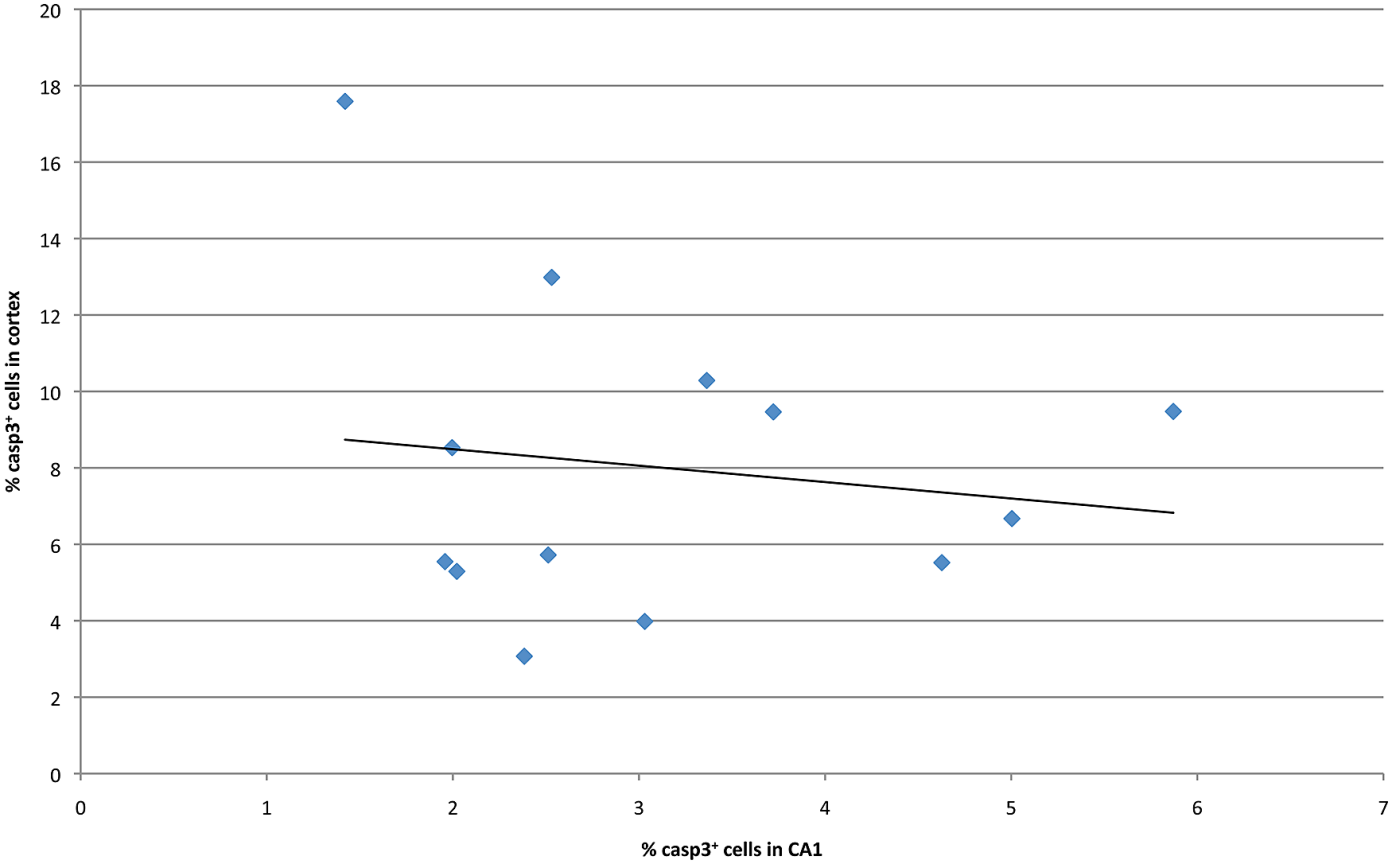
**Cell death in CA1 is plotted against averaged cell death in the cortex at P7 for all strains examined**.

Previous studies have examined malformations in various strains of mice following ethanol exposure, subsequently identifying the chromosomal locations that modulated these strain differences (Downing et al., 2012a). In order to identify potential genetic drivers of variable ethanol vulnerability, QTL analysis was performed to establish correlations between cell death levels and the genotypes across BXD lines. First, levels of caspase-3 positive cells in the hippocampus and cerebral cortex were compared in order to identify a relationship between region-specific apoptosis and genetic background. However, in the examined BXD lines, no significant correlation was observed between mean levels of ethanol-induced cell death in these regions (**Figure 4**). Thus, the hippocampus and cerebral cortex were treated as separate entities for subsequent QTL analyses.

**FIGURE 5:**
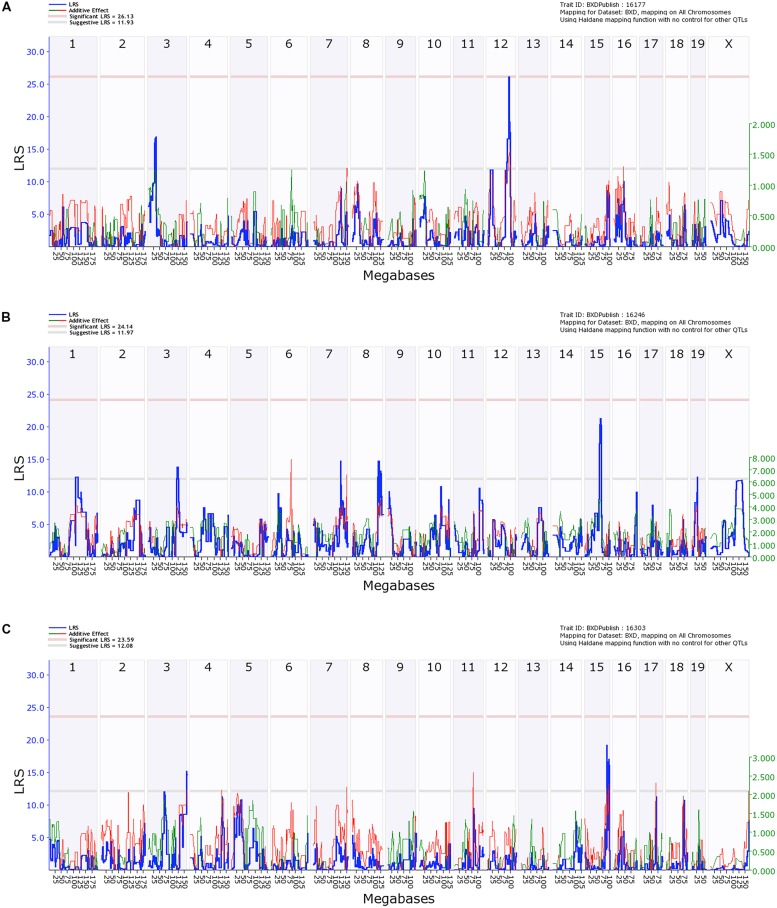
**Quantitative trait locus (QTL) analysis of cell death in the Hippocampus and Cortical Layers 2/3 and 5.** On the X-axis are the chromosomes from 1 to 19 and the X. On the Y-axis are the LRS scores. The region bounded by the gray and red horizontal lines are suggestive QTLs while the region above the red line indicates significance. **(A)** Hippocampal cell death has a significant QTL on distal Chr 12 and a suggestive QTL on proximal Chr 2. **(B)** Cell death in Layers 2/3 of cortex has a series of suggestive QTLs as does Layer 5 **(C)**.

Quantitative trait locus analysis of caspase-3 positive cells in the hippocampal CA1 region revealed a significant locus on chromosome 12 (91.0–92.3 Mb, *p* = 0.05, LRS > 26.13), which is implicated in susceptibility to ethanol-induced cell death in the hippocampus (**Figure 5A**). Genes located within the locus on chromosome 12 include Dio2 deiodinase and a number of RIKEN cDNAs.

In contrast, QTL analysis of cell death in the cerebral cortex revealed a number of suggestive loci (LRS > 12.16), though none were significant (**Figures 5B,C**). QTL analysis for Layer 5 was performed separately and only identified a suggestive locus on chromosome 3 and two loci on chromosome 15 (**Figure 5C**). No suggestive loci overlapped between the different layers of the cortex, and neither had similarities with the hippocampal CA1 region.

### ETHANOL EXPOSURE ALTERED H2A.X PHOSPHORYLATION

Given that both ethanol exposure and apoptosis are linked to the chromatin structure, we next examined their intersection on two physiologically relevant histone marks. An initial examination was conducted to assess the contribution of chromatin-based changes to cell death by measuring histone modification levels in the cerebral cortex of male P7 C57/BL6 mice treated with ethanol or saline. Two different modifications were examined to ascertain whether or not ethanol-exposure ubiquitously affects histone marks and if it alters modifications related to apoptosis. The first, phosphorylation of serine 139 on H2A.X (γH2A.X), was chosen due to its direct correlation with DNA damage and apoptosis. This histone H2A variant becomes locally phosphorylated in response to DNA damage to produce γ-H2AX foci in the vicinity of double-stranded breaks (Rogakou et al., 1998). While it does not play an active role in apoptosis, the generation of γ-H2AX during DNA fragmentation is essential for subsequent apoptotic phosphorylation of H2B (Rogakou et al., 2000; Fernandez-Capetillo et al., 2004). Thus, this modification provides a quantitative measure of ethanol-induced DNA damage, which may subsequently lead to cell death. The second, acetylation of lysine 14 on histone H3 (H3K14ace), was selected due to its association with transcriptional activation and presence in active enhancers, which may be indicative of changes in apoptotic gene expression (Karmodiya et al., 2012). A more recent study also showed that ethanol exposure increases levels of this mark in exon 1 of G9a, a histone demethylase involved in alcohol-induced apoptosis (Subbanna et al., 2014).

**FIGURE 6:**
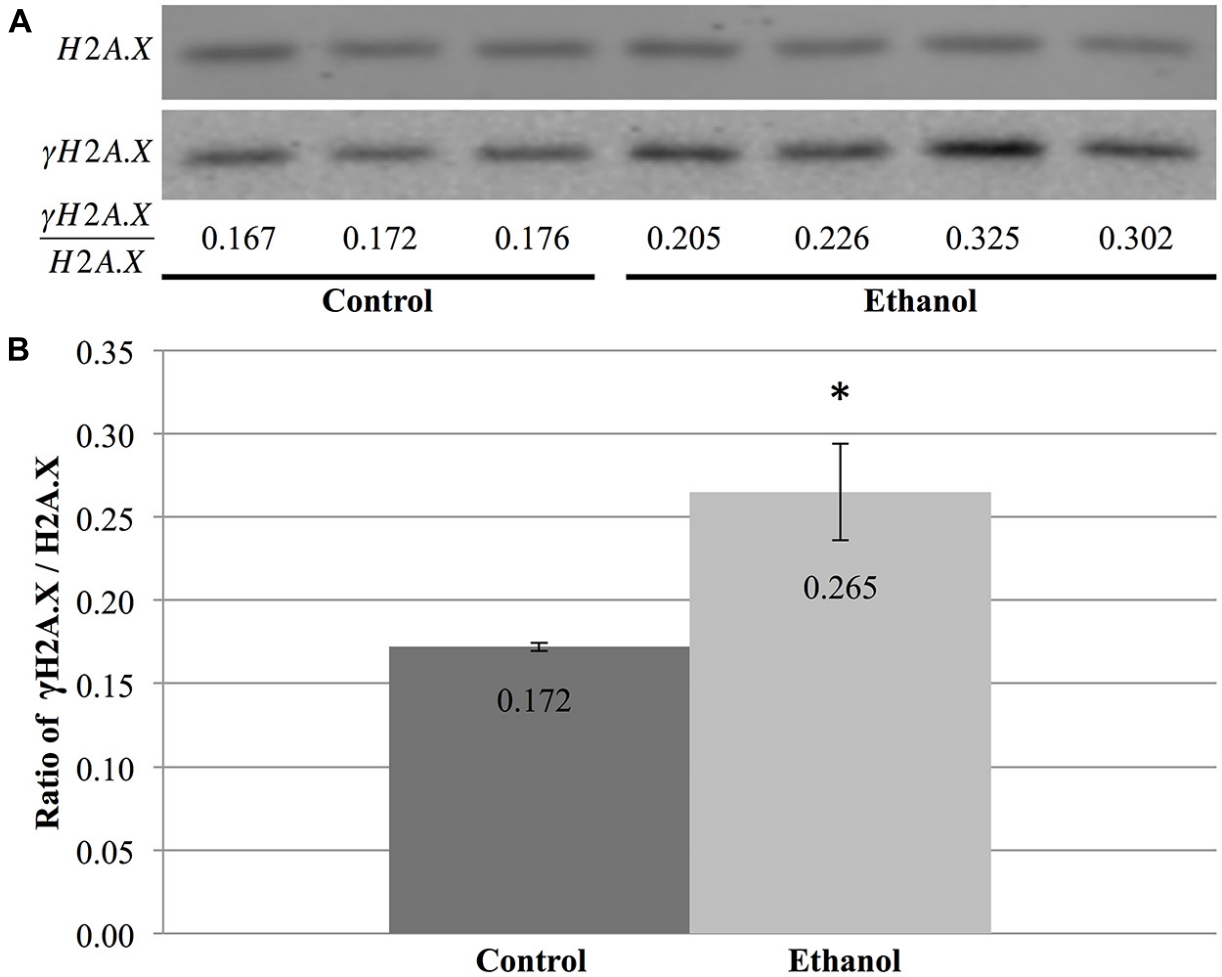
**Ethanol exposure increases levels of γH2A.X in the cerebral cortex. (A)** Protein blot analysis was performed on nuclear histones isolated from the cerebral cortex of three control and four ethanol male P7 mice. H2A.X levels were used as a loading control to normalize γH2A.X levels between samples and obtain the ratio of γH2A.X/H2A.X. **(B)** The ratio of γH2A.X to H2A.X was higher in ethanol-treated animals than in control animals (*p = 0.04). The graph is plotted as mean +/- SEM.

Using protein blots, analysis of γH2A.X in the cerebral cortex revealed a stark difference between ethanol- and saline-treated animals (**Figure 6A**). Alcohol exposure significantly increased the ratio of γH2A.X/H2A.X in the cerebral cortex when compared to saline-treated animals (*p* = 0.04; **Figure 6B**). This change in ethanol-treated animals was equivalent to a 1.54-fold increase compared to controls and was indicative of increased double-stranded breaks in DNA following ethanol exposure. Moreover, initial results also hinted at an increased γH2A.X/H2A.X ratio in both the cerebellum and hippocampus of P7 mice following acute ethanol exposure (data not shown).

**FIGURE 7:**
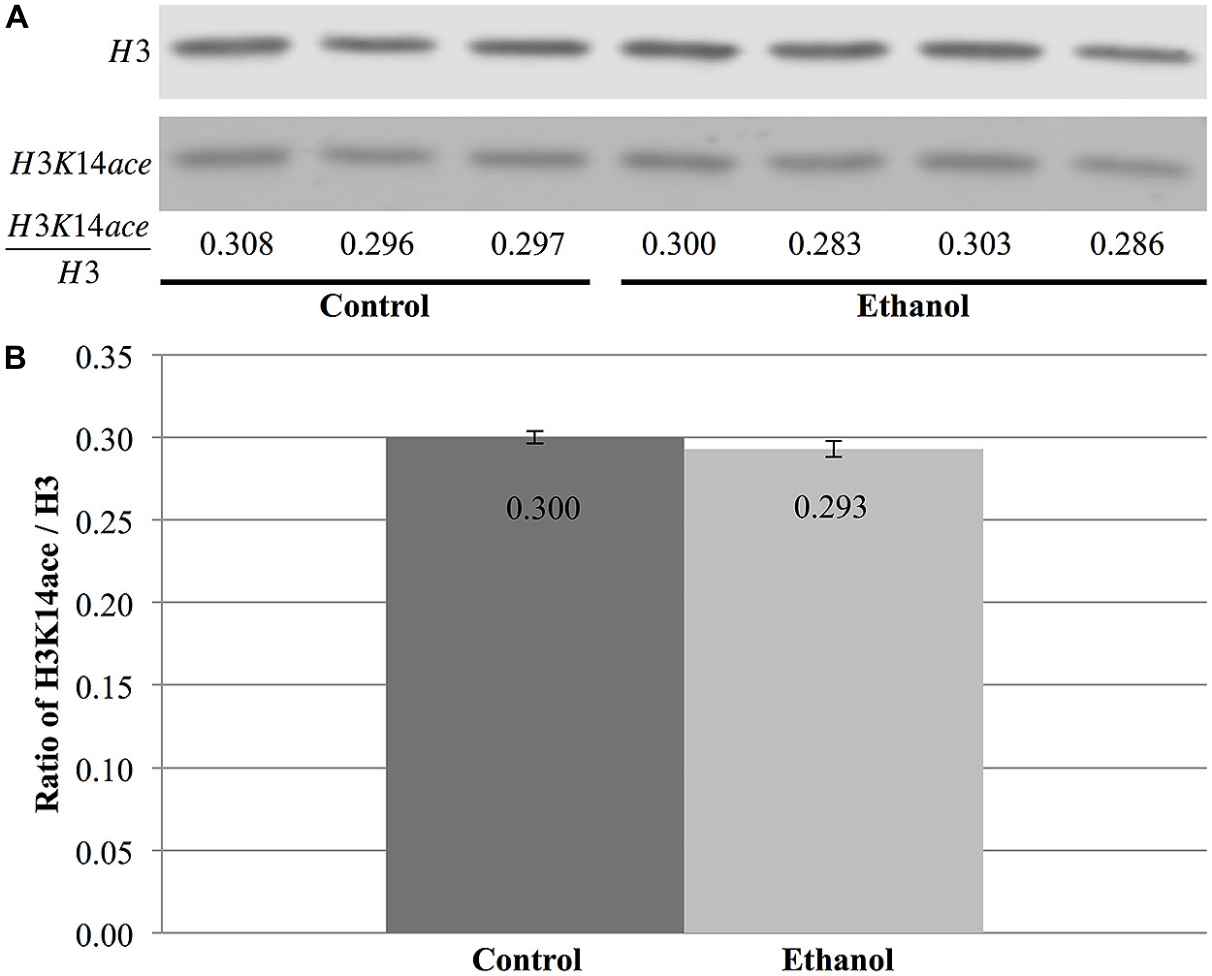
**Ethanol exposure does not affect levels of H3K14 acetylation in the cerebral cortex. (A)** Protein blot analysis was performed on nuclear histones isolated from the cerebral cortex of three control and four ethanol male P7 mice. Total H3 levels were used as a loading control to normalize H3K14 acetylation levels between samples and obtain the ratio of H3K14ace/H3. **(B)** The ratio of H3K14ace to H3 was the same in ethanol-treated animals versus control animals (*p* = 0.32). The graph is plotted as mean +/- SEM.

In contrast, total nuclear H3 and H3K14 acetylation were quantified to obtain the ratio of H3K14ace/H3, which is indicative of the relative amount of acetylated H3K14 (**Figure 7A**). However, this analysis did not reveal any differences between saline and ethanol-treated animal (**Figure 7A**), as the ratio of H3K14ace/H3 was almost identical for both treatment groups (*p* = 0.32, **Figure 7B**).

## DISCUSSION

In the present study, lines of recombinant inbred mice were examined to determine if genetic variation influences the extent and localization of ethanol-induced cell death in the developing cerebral cortex and hippocampus at P7. Differential strain sensitivity to ethanol was observed, with hippocampal data translating into the identification of a QTL on chromosome 12, which mediates these strain-specific differences. To assess whether acute ethanol exposure also causes chromatin-based alterations in the developing brain, two histone modifications were examined in the cerebral cortex. Interestingly, acute ethanol exposure increased levels of γH2AX, a histone mark associated with DNA fragmentation, which is characteristic of apoptosis.

Several studies have previously examined cell death in the developing cerebral cortex and hippocampus following neonatal ethanol exposure in both rats and mice (Olney et al., 2002a,b; Wozniak et al., 2004; Young and Olney, 2006). Several of these studies utilized the same alcohol exposure paradigm and one of the same strains, B6, as the present study, which allows for direct comparisons between our results and published data. While Olney et al. (2002a,b) examined more global changes due to ethanol, rather than focusing on specific regions within structures, it is interesting to note that these papers also showed high levels of cell death within the CA1 region of the hippocampus and in much of the cerebral cortex. The type of cell death in the hippocampus has also been examined and all studies to date have shown evidence that these cells are dying via apoptotic mechanisms (e.g., Olney et al., 2002a; Wozniak et al., 2004; Young et al., 2005; Young and Olney, 2006; Ullah et al., 2011) consistent with the present study.

Additionally, examination of neuron number following neonatal ethanol exposure has been examined in the hippocampus. Because hippocampal neurons are generated prenatally, any decrease in neuronal number must result from cell death. These studies demonstrate that, within the hippocampus, the CA1 cells are highly susceptible to ethanol-induced cell death following exposure during the brain growth spurt (Livy et al., 2003; Tran and Kelly, 2003; de Licona et al., 2009). These studies are consistent with the results of the present study.

A caveat of the present experiment is that the type of cell that is undergoing apoptosis is unknown. Ethanol has been shown to result in cell death in both neurons and glia (e.g., Guerri et al., 2001; Dikranian et al., 2005) suggesting that either population could be the target. However, the location and morphology of the developing cells support the hypothesis that neurons are the vulnerable cell population in this ethanol exposure paradigm. The identification of the vulnerable population can provide insights to the identification of susceptibility genes that underlie the QTL and this will be examined in further studies using this model.

One of the issues in the present study is the small number of strains and how that may impact the ability to detect significant QTLs as well as the reliability of the significant QTL that was detected. It is well known that the ability to detect QTLs is strongly influenced by the number of genes involved such that when there are a small number of genes with large effects on the phenotypic outcome, significant QTLs can be detected with a low number of strains (Flint et al., 2005). In the present experiment, a significant QTL was detected in the hippocampus suggesting that there is a gene within the QTL that has a large impact on genetic differences in ethanol-induced neurodegeneration in that brain region. In contrast, the small number of strains used in the present study translates into lower statistical power that can mean that significant QTLs are not detected. In the cerebral cortex in the present experiment, this is the case and if more strains had been examined, significant, rather than suggestive, QTLs may have been identified in the cortex as well. In regards to the issue of the reliability of significant QTLs, the presence of outliers is an important consideration. Confidence in the validity of a QTL is lessened if there are outliers that are strongly impacting the strain distribution in the phenotypic readout (Hayat et al., 2008; Yang et al., 2009). In the present experiment, the strains show a relatively even distribution and therefore, outliers are not influencing the current analyses providing support that this is a reliable QTL.

Quantitative trait locus analysis demonstrated that the strain-specific differences in ethanol-induced cell death in the hippocampus are modulated, at least in part, by a gene located on Chromosome 12 at approximately 90 Mb. This is a novel chromosomal location with no previously established relationship to ethanol’s teratogenic actions (Downing et al., 2012a). This region is relatively narrow with only two genes, six RIKEN clones, and one EST located within the QTL interval. Cell death, and in particular apoptosis, can be caused by a number of mechanisms including loss of growth factors and excitotoxicity (e.g., Bhutta and Anand, 2002; Nikolić et al., 2013). It is of interest therefore, that proximal to the region of the QTL there are several genes that are related to growth factors including the latent transforming growth factor protein 2 (ltbp2), placental growth factor (pgf), and transforming growth factor beta (Tgf beta). However, while these are certainly interesting candidates that cannot be excluded at this time, the rapid nature of the cell death induction in the present analysis led to the hypothesis that the causal gene underlying the QTL is more likely to have a direct link to cell death and the analysis was focused on these genes.

This region of the genome contained several genes that were more directly linked to apoptosis and cell death. However, all the potential candidate genes have the following caveats: (1) none has been linked to cell death following any form of alcohol exposure, and (2) while present in the vicinity of the Ch 12 QTL, none are within the 1LOD interval of the QTL. The candidate gene with the strongest link to apoptosis is Rbm25, an RNA binding protein that has been shown to modulate the expression of isoforms of Bcl2 (Zhou et al., 2008). However, Rbm25 is located farthest from the QTL and its role in apoptosis within the CNS remains unknown. Additionally, both fos and the fos receptor (fosr) as well as jun dimerization protein (jdp2 or jund2) are located close to the QTL. While Fos and jdp2 have been linked to apoptosis (e.g., Lerdrup et al., 2005; Durchdewald et al., 2009), these intermediate early genes have well-documented functions in a number of processes and thus, the specificity of the effects to the apoptotic processes is currently unknown.

Based on the current data, the best candidate is neuroglobin (Ngb), a relatively recently described gene that encodes a protein that functions to provide oxygen to the CNS (Fiocchetti et al., 2013). Ngb sits close to the QTL region and, as shown on WebQTL, possesses several sequence polymorphisms between the B6 and D2 genome. Genes that lack sequence polymorphisms, and therefore are identical between the two strains, are less likely to mediate strain differences. Moreover, Ngb has been linked to apoptosis caused by a range of factors including oxidative stress (Li et al., 2008) and arsenic toxicity (Liu et al., 2013) while also playing a neuroprotective role following stroke (Yu et al., 2013). Given that hypoxia has been suggested to play a role in ethanol’s teratogenic effects (Mukherjee and Hodgen, 1982; Mitchell et al., 1998; Parnell et al., 2007), neuroglobulin is an intriguing candidate for mediating strain differences in ethanol-induced cell death.

Differences in chromatin regulation may also be contributing to the strain-specific differences in cell death. Epigenetic marks are emerging as major regulators of gene-by-environment interactions and have been implicated in the etiology of ethanol-induced neurodegeneration. In fact, G9a-mediated increases of H3K9 and H3K27 dimethylation regulate proteolytic cleavage of histones by caspase-3 and subsequent neurodegeneration in the hippocampus and neocortex following acute, low-dose ethanol exposure (Subbanna et al., 2013). Furthermore, the same group recently showed H3K14 acetylation levels on exon1 of G9a increase following low-dose ethanol exposure (Subbanna et al., 2014).

Our results also support a role for chromatin-based marks in ethanol-induced apoptosis of cells in the CNS. However, similar to previous studies examining cocaine exposure (Jordi et al., 2013), this remains a global analysis of the genome, rather than a targeted gene approach, and does not identify changes in H3K14 acetylation or γH2A.X in specific regions of the genome. While the cerebral cortex did not show any differences in the levels of H3K14 acetylation following alcohol exposure, the ratio of γH2A.X to H2A.X was increased by an acute ethanol treatment. Although these two marks do not perform the same function, these results suggest that ethanol does not have a broad impact over different histone modifications, but, rather, acts in a specific fashion by altering at least a subset of chromatin-based mechanisms linked to apoptosis. Moreover, this effect seemed to occur across all analyzed brain regions (cortex; preliminary data: cerebellum, hippocampus), implying that this is a global response to ethanol exposure, rather than a region-specific event.

The observed difference of γH2A.X in the cerebral cortex following alcohol exposure is likely caused by increased rates of double-stranded breaks (Rogakou et al., 2000). This may be a direct effect of ethanol, where its exposure increases reactive oxygen species (ROS) levels in the cell, which, in turn, cause more DNA damage (Kotch et al., 1995). Widespread DNA damage might then cause the affected cells to undergo apoptosis. In this situation, γH2A.X might play a mechanistic role in ethanol-induced neuroapoptosis. However, the effect may also be indirect, where, instead, ethanol exposure activates apoptotic programming through other mechanisms, leading to DNA fragmentation and subsequent H2A.X phosphorylation (Rogakou et al., 2000). Here, γH2A.X would simply be a passenger to neurodegeneration, a simple consequence of cell death. Though there is evidence in the field for both these possibilities, the current data does not allow for a distinction to be made between the two. However, further experiments investigating the link between ROS and γH2A.X following ethanol exposure and the activation patterns of apoptotic programs may provide additional insight into their plausibility.

Similar to the issue discussed above, protein blot analysis of the whole cerebral cortex does not allow for differentiation between the types of cells affected by ethanol. As the brain is composed of many different cell types, unaffected cells may dampen some signal in the event that a single cellular species is affected by ethanol in this fashion. In turn, this would explain the relatively low fold change of ethanol-treated animals compared to control (1.54). Higher resolution techniques, such as immunofluorescence will be better suited for this type of analysis in additional mouse strains.

Several studies have also begun to characterize DNA methylation changes following prenatal and postnatal ethanol exposure (Haycock, 2009; Haycock and Ramsay, 2009). In mouse models, ethanol exposure during embryonic days 9–11 was shown to cause global genomic hypomethylation and decreased DNA methyltransferase activity in the fetal genome (Garro et al., 1991). However, recent studies have shown that some regions become hypermethylated and others hypomethylated in embryonic cultures exposed to ethanol (Liu et al., 2009; Chen et al., 2013). In vivo animal studies have corroborated these findings, showing that fetal alcohol exposure results in long-lasting alteration to the brain’s DNA methylome, notably in regulatory sequences and imprinted regions containing non-coding RNA (Laufer et al., 2013). Combined with data on histone modifications, these studies support a role for epigenetic changes in ethanol-induced developmental defects.

In summary, we demonstrate that acute neonatal ethanol exposure causes cell death in the developing cerebral cortex and hippocampus in a strain-specific manner. Moreover, we show that a region on chromosome 12 could, at least in part, mediate differential strain sensitivity to ethanol in the hippocampus and identify a number of putative candidate genes that may underlie this QTL. Further studies are required to pinpoint the gene(s) that mediates this vulnerability to ethanol-induced apoptosis in the hippocampus, as well as other brain regions. Identification will be facilitated by identifying whether any genes within the QTL exhibit strain-specific changes in expression following ethanol exposure similar to other studies examining stain-specific expression differences (Green et al., 2007; Downing et al., 2012b). This study also begins to examine whether chromatin-based modifications contribute to differential strain sensitivity, showing that acute ethanol exposure can indeed alter histone modifications. Additional studies into the level of epigenetic alteration between different genotypes will be required to identify their role in variable susceptibility to ethanol-induced neurodegeneration.

## Conflict of Interest Statement

The authors declare that the research was conducted in the absence of any commercial or financial relationships that could be construed as a potential conflict of interest.
